# (Penta­fluoro­propionato-κ*O*)tetra­kis­(trimethyl­phosphine oxide-κ*O*)copper(II) penta­fluoro­propionate

**DOI:** 10.1107/S1600536811031114

**Published:** 2011-08-11

**Authors:** Iwona B. Szymańska, Liliana Dobrzańska

**Affiliations:** a Faculty of Chemistry, Nicolaus Copernicus University, Gagarina 7,87-100 Toruń, Poland; b Department of Chemistry, Katholieke Universiteit Leuven, Celestijnenlaan 200F - bus 2404, B-3001 Heverlee, Belgium; c Department of Chemistry, University of Stellenbosch, Private Bag X1, Matieland, South Africa

## Abstract

The title compound, [Cu(C_3_F_5_O_2_)(C_3_H_9_OP)_4_](C_3_F_5_O_2_), comprises a cationic Cu^II^ complex and a disordered penta­fluoro­propionate counter-ion. The metal atom has a distorted square-pyramidal coordination environment formed by four O atoms originating from trimethyl­phosphine oxide mol­ecules and the remaining one belonging to the monodentate penta­fluoro­propionate anion, which is situated in the basal plane of the pyramid. The mol­ecules are held together in the crystal by a net of weak C—H⋯O and C—H⋯F hydrogen bonds. The counter anion is disordered over two sets of sites in a 0.629 (5):0.371 (5) ratio.

## Related literature

For our previous studies on metal complexes suitable for chemical vapour deposition (CVD), see: Szymańska *et al.* (2007 ▶, 2009 ▶); Piszczek *et al.* (2008 ▶). For crystal structures of metal complexes with trimethyl­phosphine oxide ligands involving metal ions from the first transition series, see: Hill *et al.* (2003 ▶) for Sc(III); Johnson & Bergman (2001 ▶) for Ti(III); Veige *et al.* (2003 ▶) for V(III); Cotton *et al.* (1991 ▶) for Fe(II); Edelmann & Behrens (1986 ▶) for Co(II); Klein *et al.* (1999 ▶) for Ni(II); Hlavinka & Hagadorn (2005 ▶) for Zn(II). For crystallographic data on Cu^II^ complexes with a penta­fluoro­propionate ligand, see: Jiang *et al.* (1998 ▶); Zhang *et al.* (1999 ▶).
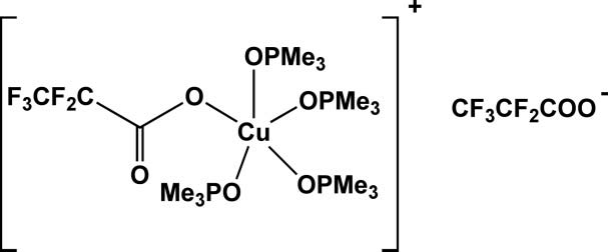


## Experimental

### 

#### Crystal data

[Cu(C_3_F_5_O_2_)(C_3_H_9_OP)_4_](C_3_F_5_O_2_)
*M* *_r_* = 757.89
Triclinic, 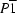

*a* = 9.5955 (8) Å
*b* = 12.2627 (11) Å
*c* = 14.1848 (12) Å
α = 82.720 (2)°
β = 80.501 (1)°
γ = 82.899 (2)°
*V* = 1623.9 (2) Å^3^
*Z* = 2
Mo *K*α radiation
μ = 0.96 mm^−1^
*T* = 100 K
0.48 × 0.17 × 0.03 mm

#### Data collection

Bruker APEX CCD area-detector diffractometer
Absorption correction: multi-scan (*SADABS*; Sheldrick, 1997 ▶) *T*_min_ = 0.655, *T*_max_ = 0.972
10254 measured reflections
7148 independent reflections
6207 reflections with *I* > 2σ(*I*)
*R*_int_ = 0.016

#### Refinement

*R*[*F* ^2^ > 2σ(*F* ^2^)] = 0.047
*wR*(*F* ^2^) = 0.124
*S* = 1.07
7148 reflections
416 parameters
33 restraints
H-atom parameters constrained
Δρ_max_ = 1.33 e Å^−3^
Δρ_min_ = −0.64 e Å^−3^

### 

Data collection: *SMART* (Bruker, 2001 ▶); cell refinement: *SAINT* (Bruker, 2002 ▶); data reduction: *SAINT*; program(s) used to solve structure: *SHELXS97* (Sheldrick, 2008 ▶); program(s) used to refine structure: *SHELXL97* (Sheldrick, 2008 ▶); molecular graphics: *Mercury* (Macrae *et al.*, 2008 ▶); software used to prepare material for publication: *SHELXL97*.

## Supplementary Material

Crystal structure: contains datablock(s) I, global. DOI: 10.1107/S1600536811031114/hp2011sup1.cif

Structure factors: contains datablock(s) I. DOI: 10.1107/S1600536811031114/hp2011Isup2.hkl

Additional supplementary materials:  crystallographic information; 3D view; checkCIF report

## Figures and Tables

**Table:** 

Cu1—O1	1.9535 (18)
Cu1—O4	1.9582 (18)
Cu1—O3	1.965 (2)
Cu1—O5	1.9863 (19)
Cu1—O2	2.1876 (19)

**Table:** 

O1—Cu1—O4	172.44 (8)
O1—Cu1—O3	90.48 (8)
O4—Cu1—O3	88.42 (8)
O1—Cu1—O5	91.12 (8)
O4—Cu1—O5	87.71 (8)
O3—Cu1—O5	162.51 (8)
O1—Cu1—O2	94.54 (7)
O4—Cu1—O2	92.99 (7)
O3—Cu1—O2	102.55 (8)
O5—Cu1—O2	94.69 (8)

**Table 2:** Geometry of selected hydrogen bonds (Å, °)

*D*—H⋯*A*	*D*—H	H⋯*A*	*D*⋯*A*	*D*—H⋯*A*
C3—H3*B*⋯O5	0.98	2.57	3.265 (3)	128
C5—H5*A*⋯O4	0.98	2.42	3.204 (4)	136
C10—H10*B*⋯O3	0.98	2.58	3.266 (4)	127
C1—H1*A*⋯F2^i^	0.98	2.52	3.392 (3)	149
C1—H1*B*⋯O6^ii^	0.98	2.47	3.265 (3)	138
C4—H4*A*⋯F4^iii^	0.98	2.51	3.482 (3)	170
C4—H4*C*⋯F3^i^	0.98	2.53	3.478 (5)	163
C9—H9*A*⋯F7*A*	0.98	2.44	3.322 (6)	150
C11—H11*B*⋯O8*A*^iv^	0.98	2.42	3.289 (7)	147
C12—H12*B*⋯O8*A*^iv^	0.98	2.52	3.388 (8)	147

Symmetry codes: (i) 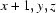; (ii) 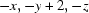; (iii) 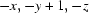; (iv) 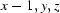.

**Fig. 1.**
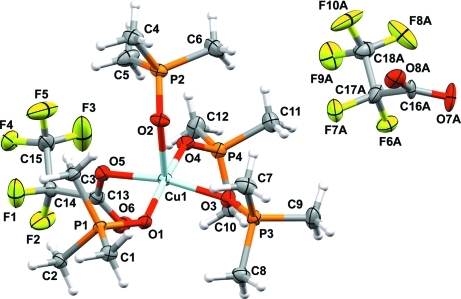
The molecular structure of the title compound, with the atom-numbering scheme. Displacement ellipsoids are drawn at the 50% probability level; the other orientation of the disordered counter-ion has been omitted for clarity.

## supplementary crystallographic information

### Comment

During our ongoing studies on metal complexes with tertiary phosphines (Szymańska *et al.*, 2007) and perfluorinated carboxylates (Piszczek *et al.*, 2008; Szymańska *et al.*, 2009) suitable for chemical vapour deposition (CVD), the title compound was accidentally isolated. It is the first report on the crystal structure of a Cu complex with trimethylphosphine oxides. There is however some literature on coordination compounds of trimethylphosphine oxide ligands with other metals from the first transition series such as: Sc(III) (Hill *et al.*, 2003), Ti(III) (Johnson & Bergman, 2001), V(III) (Veige *et al.*, 2003), Fe(II) (Cotton *et al.*, 1991), Co(II) (Edelmann & Behrens, 1986), Ni(II) (Klein *et al.*, 1999), Zn(II) (Hlavinka & Hagadorn, 2005).

Furthermore, there are also only two reports on crystal structures of Cu^II^ complexes containing coordinating pentafluoropropionate ions (Jiang *et al.*, 1998; Zhang *et al.*, 1999).

The title compound has one monocationic Cu^II^ complex and one pentafluoropropionate counter-ion present in the asymmetric unit (Fig.1). The geometry around the Cu^II^ ion is a distorted square-pyramid formed by four O atoms originating from trimethylphosphine oxide molecules and one from the monodentate pentafluoropropionate ion, which is located in the base plane of the pyramid. The corresponding bond lengths and angles are presented in Table 1. The geometrical features of the ligands are in good agreement with reported values. The counter-ion is disordered over two positions with refined site occupancies of 0.629 (5):0.371 (5). There are weak intramolecular C—H···O interactions between the methyl groups of three distinct trimethylphosphine oxides (P1, P2 and P4) involving the atoms C3, C5 and C10, that act as donors, and O5 (from the counter-ion), as well as O4 and O3 (from the oxide ligands), that act as acceptors, respectively (Table 2). The C9 methyl group from P3 however, interacts with the counter-ion by weak C9—H9A···F7A hydrogen bonding with a C···F distance of 3.322 (6) Å, and a C—H—F angle of 150°. The packing is further stabilized by numerous weak intermolecular C—H···O and C—H···F interactions.

### Experimental

(C_2_F_5_COO)_2_Cu (1.04 mmol) was placed in a Schlenk tube, dissolved in 25 ml of freshly distilled acetonitrile, and copper powder (5 mmol) was added. The obtained suspension was stirred until the solution was pale yellow. Then PMe_3_ (2.1 ml of a 1 *M* THF solution) was added and the reaction mixture was stirred for 18 h at ambient temperature, and filtered. The solvent was evaporated under reduced pressure, yielding [Cu_2_(PMe_3_)_2_(µ–C_2_F_5_CO_2_)_2_] as a pale yellow, viscous oil. Crystals of the title Cu^II^ complex suitable for X-ray studies were obtained after a few months, presumably upon slow oxidation by diffused air.

### Refinement

All H atoms were positioned geometrically, with C—H = 0.98 and constrained to ride on their parent atoms with *U*_iso_(H) = 1.5*U*_eq_(C). The counter-ion was found to be disordered and modeled in two positions. Refinement included bond lengths restraints applied to the O8B—O7B, C17B—O7B and C17B—O8B as well as to ADPs of 'A' part.

### Crystal data

**Table:**

[Cu(C_3_F_5_O_2_)(C_3_H_9_OP)_4_](C_3_F_5_O_2_)	*Z* = 2
*M**_r_* = 757.89	*F*(000) = 774
Triclinic, *P*1	*D*_x_ = 1.550 Mg m^−^^3^
Hall symbol: -P 1	Mo *K*α radiation, λ = 0.71073 Å
*a* = 9.5955 (8) Å	Cell parameters from 5155 reflections
*b* = 12.2627 (11) Å	θ = 2.2–28.1°
*c* = 14.1848 (12) Å	µ = 0.96 mm^−^^1^
α = 82.720 (2)°	*T* = 100 K
β = 80.501 (1)°	Plate, colorless
γ = 82.899 (2)°	0.48 × 0.17 × 0.03 mm
*V* = 1623.9 (2) Å^3^	

### Data collection

**Table:**

Bruker APEX CCD area-detector diffractometer	7148 independent reflections
Radiation source: fine-focus sealed tube	6207 reflections with *I* > 2σ(*I*)
graphite	*R*_int_ = 0.016
ω scans	θ_max_ = 28.3°, θ_min_ = 2.4°
Absorption correction: multi-scan (*SADABS*; Sheldrick, 1997)	*h* = −10→12
*T*_min_ = 0.655, *T*_max_ = 0.972	*k* = −12→15
10254 measured reflections	*l* = −12→18

### Refinement

**Table:**

Refinement on *F*^2^	Primary atom site location: structure-invariant direct methods
Least-squares matrix: full	Secondary atom site location: difference Fourier map
*R*[*F*^2^ > 2σ(*F*^2^)] = 0.047	Hydrogen site location: inferred from neighbouring sites
*wR*(*F*^2^) = 0.124	H-atom parameters constrained
*S* = 1.07	*w* = 1/[σ^2^(*F*_o_^2^) + (0.0677*P*)^2^ + 1.8569*P*] where *P* = (*F*_o_^2^ + 2*F*_c_^2^)/3
7148 reflections	(Δ/σ)_max_ = 0.004
416 parameters	Δρ_max_ = 1.33 e Å^−^^3^
33 restraints	Δρ_min_ = −0.64 e Å^−^^3^

### Special details

**Table:**

Geometry. All e.s.d.'s (except the e.s.d. in the dihedral angle between two l.s. planes) are estimated using the full covariance matrix. The cell e.s.d.'s are taken into account individually in the estimation of e.s.d.'s in distances, angles and torsion angles; correlations between e.s.d.'s in cell parameters are only used when they are defined by crystal symmetry. An approximate (isotropic) treatment of cell e.s.d.'s is used for estimating e.s.d.'s involving l.s. planes.
Refinement. Refinement of *F*^2^ against ALL reflections. The weighted *R*-factor *wR* and goodness of fit *S* are based on *F*^2^, conventional *R*-factors *R* are based on *F*, with *F* set to zero for negative *F*^2^. The threshold expression of *F*^2^ > σ(*F*^2^) is used only for calculating *R*-factors(gt) *etc*. and is not relevant to the choice of reflections for refinement. *R*-factors based on *F*^2^ are statistically about twice as large as those based on *F*, and *R*- factors based on ALL data will be even larger.

### Fractional atomic coordinates and isotropic or equivalent isotropic displacement parameters (Å^2^)

**Table:**

	*x*	*y*	*z*	*U*_iso_*/*U*_eq_	Occ. (<1)
Cu1	0.08108 (3)	0.78725 (2)	0.19051 (2)	0.01610 (10)	
P1	0.24449 (7)	0.85300 (5)	−0.01852 (5)	0.01593 (14)	
F1	−0.1350 (2)	0.6864 (2)	−0.05341 (14)	0.0504 (6)	
O1	0.18617 (19)	0.87540 (15)	0.08413 (13)	0.0192 (4)	
C1	0.3916 (3)	0.9301 (2)	−0.0631 (2)	0.0219 (5)	
H1B	0.3621	1.0088	−0.0583	0.033*	
H1C	0.4253	0.9186	−0.1305	0.033*	
H1A	0.4683	0.9053	−0.0251	0.033*	
P2	0.25316 (7)	0.53881 (6)	0.24912 (5)	0.02095 (16)	
F2	−0.3344 (2)	0.76050 (17)	0.0154 (2)	0.0551 (7)	
O2	0.2545 (2)	0.65462 (16)	0.20071 (14)	0.0241 (4)	
C2	0.1173 (3)	0.8928 (2)	−0.09896 (19)	0.0216 (5)	
H2A	0.0808	0.9706	−0.0950	0.032*	
H2C	0.0386	0.8467	−0.0810	0.032*	
H2B	0.1628	0.8829	−0.1649	0.032*	
P3	0.23148 (7)	0.95789 (6)	0.28343 (5)	0.02113 (16)	
F3	−0.3038 (4)	0.5932 (3)	0.17037 (17)	0.0865 (11)	
O3	0.1153 (2)	0.88590 (17)	0.28130 (14)	0.0239 (4)	
C3	0.3067 (3)	0.7111 (2)	−0.0298 (2)	0.0241 (6)	
H3B	0.2287	0.6654	−0.0061	0.036*	
H3C	0.3841	0.6884	0.0081	0.036*	
H3A	0.3416	0.7017	−0.0975	0.036*	
P4	−0.15738 (7)	0.75219 (6)	0.37572 (5)	0.02040 (16)	
F4	−0.3406 (2)	0.54896 (16)	0.03595 (14)	0.0372 (4)	
O4	−0.0456 (2)	0.71361 (15)	0.29524 (14)	0.0219 (4)	
C4	0.4003 (3)	0.4476 (3)	0.2000 (2)	0.0311 (7)	
H4C	0.4893	0.4776	0.2029	0.047*	
H4A	0.3931	0.4405	0.1330	0.047*	
H4B	0.3988	0.3747	0.2374	0.047*	
F5	−0.1333 (3)	0.51630 (18)	0.0786 (2)	0.0734 (9)	
O5	−0.00828 (19)	0.71780 (16)	0.09965 (14)	0.0210 (4)	
C5	0.0986 (3)	0.4756 (3)	0.2401 (3)	0.0339 (7)	
H5A	0.0134	0.5217	0.2659	0.051*	
H5C	0.1016	0.4023	0.2769	0.051*	
H5B	0.0959	0.4681	0.1724	0.051*	
O6	−0.1970 (2)	0.84408 (17)	0.12865 (17)	0.0327 (5)	
C6	0.2642 (4)	0.5320 (3)	0.3743 (2)	0.0373 (8)	
H6A	0.1857	0.5805	0.4053	0.056*	
H6B	0.3548	0.5561	0.3822	0.056*	
H6C	0.2581	0.4558	0.4040	0.056*	
C7	0.4043 (3)	0.8860 (3)	0.2605 (2)	0.0337 (7)	
H7B	0.4105	0.8184	0.3052	0.050*	
H7C	0.4752	0.9333	0.2695	0.050*	
H7A	0.4224	0.8668	0.1943	0.050*	
C8	0.2270 (3)	1.0803 (2)	0.2011 (2)	0.0285 (6)	
H8B	0.1326	1.1216	0.2115	0.043*	
H8A	0.2473	1.0603	0.1351	0.043*	
H8C	0.2987	1.1262	0.2113	0.043*	
C9	0.2105 (4)	1.0049 (3)	0.3994 (2)	0.0371 (8)	
H9B	0.1156	1.0445	0.4138	0.056*	
H9C	0.2826	1.0547	0.4003	0.056*	
H9A	0.2218	0.9413	0.4479	0.056*	
C10	−0.2257 (3)	0.8943 (3)	0.3576 (2)	0.0297 (6)	
H10B	−0.1475	0.9407	0.3488	0.045*	
H10C	−0.2944	0.9133	0.4138	0.045*	
H10A	−0.2728	0.9067	0.3003	0.045*	
C11	−0.0891 (3)	0.7320 (3)	0.4869 (2)	0.0317 (7)	
H11A	−0.0069	0.7738	0.4812	0.048*	
H11C	−0.0601	0.6532	0.5025	0.048*	
H11B	−0.1629	0.7579	0.5379	0.048*	
C12	−0.3045 (3)	0.6728 (3)	0.3907 (2)	0.0308 (7)	
H12C	−0.2711	0.5940	0.4011	0.046*	
H12A	−0.3503	0.6869	0.3329	0.046*	
H12B	−0.3730	0.6938	0.4464	0.046*	
C13	−0.1330 (3)	0.7628 (2)	0.0938 (2)	0.0221 (5)	
C14	−0.2120 (3)	0.7015 (2)	0.0332 (2)	0.0238 (6)	
C15	−0.2495 (4)	0.5889 (3)	0.0808 (2)	0.0369 (8)	
O7A	0.4474 (9)	0.8498 (7)	0.7251 (5)	0.0477 (17)	0.629 (5)
O8A	0.5882 (6)	0.7691 (6)	0.6074 (5)	0.0323 (13)	0.629 (5)
C16A	0.4755 (7)	0.8008 (5)	0.6533 (5)	0.021 (2)	0.629 (5)
C17A	0.3420 (5)	0.7754 (4)	0.6121 (4)	0.0329 (9)	0.629 (5)
F6A	0.2231 (3)	0.8483 (2)	0.6315 (2)	0.0347 (8)	0.629 (5)
F7A	0.3679 (4)	0.7818 (4)	0.5132 (2)	0.0379 (9)	0.629 (5)
C18A	0.2969 (6)	0.6623 (4)	0.6453 (4)	0.0372 (10)	0.629 (5)
F8A	0.2645 (6)	0.6562 (5)	0.7412 (3)	0.0703 (16)	0.629 (5)
F9A	0.1885 (4)	0.6422 (4)	0.6027 (4)	0.0480 (12)	0.629 (5)
F10A	0.3998 (4)	0.5841 (3)	0.6197 (4)	0.0678 (16)	0.629 (5)
O7B	0.4232 (16)	0.8398 (13)	0.7137 (9)	0.052 (4)*	0.371 (5)
O8B	0.5843 (13)	0.8011 (11)	0.5898 (11)	0.071 (6)	0.371 (5)
C16B	0.4611 (9)	0.7981 (10)	0.6411 (8)	0.035 (6)*	0.371 (5)
C17B	0.3690 (6)	0.7217 (6)	0.6021 (4)	0.041 (2)*	0.371 (5)
F7B	0.3527 (9)	0.7481 (6)	0.5070 (4)	0.053 (3)*	0.371 (5)
F6B	0.4318 (6)	0.6141 (5)	0.6041 (5)	0.0376 (18)*	0.371 (5)
C18B	0.2217 (7)	0.7160 (6)	0.6562 (4)	0.046 (2)*	0.371 (5)
F8B	0.1567 (7)	0.8193 (4)	0.6525 (4)	0.0486 (17)*	0.371 (5)
F9B	0.1530 (7)	0.6463 (5)	0.6187 (4)	0.0319 (19)*	0.371 (5)
F10B	0.2208 (7)	0.6733 (6)	0.7469 (4)	0.0378 (18)*	0.371 (5)

### Atomic displacement parameters (Å^2^)

**Table:**

	*U*^11^	*U*^22^	*U*^33^	*U*^12^	*U*^13^	*U*^23^
Cu1	0.01510 (16)	0.01617 (17)	0.01697 (17)	−0.00428 (12)	−0.00109 (11)	−0.00082 (12)
P1	0.0158 (3)	0.0150 (3)	0.0169 (3)	−0.0026 (2)	−0.0013 (2)	−0.0017 (2)
F1	0.0599 (14)	0.0691 (15)	0.0299 (10)	−0.0364 (12)	−0.0006 (9)	−0.0139 (10)
O1	0.0221 (9)	0.0172 (9)	0.0179 (9)	−0.0055 (7)	0.0004 (7)	−0.0017 (7)
C1	0.0181 (12)	0.0232 (13)	0.0236 (13)	−0.0048 (10)	−0.0008 (10)	−0.0002 (11)
P2	0.0181 (3)	0.0204 (3)	0.0220 (3)	0.0000 (3)	−0.0013 (2)	0.0024 (3)
F2	0.0481 (12)	0.0329 (11)	0.0966 (19)	0.0074 (9)	−0.0504 (13)	−0.0130 (11)
O2	0.0189 (9)	0.0232 (10)	0.0273 (10)	−0.0015 (8)	−0.0017 (8)	0.0053 (8)
C2	0.0213 (12)	0.0226 (13)	0.0213 (13)	−0.0041 (11)	−0.0044 (10)	−0.0008 (10)
P3	0.0221 (3)	0.0230 (4)	0.0199 (3)	−0.0078 (3)	−0.0044 (3)	−0.0015 (3)
F3	0.148 (3)	0.096 (2)	0.0307 (12)	−0.097 (2)	0.0034 (15)	−0.0048 (13)
O3	0.0256 (10)	0.0268 (10)	0.0204 (9)	−0.0106 (8)	0.0011 (8)	−0.0057 (8)
C3	0.0280 (14)	0.0170 (13)	0.0253 (14)	0.0011 (11)	0.0004 (11)	−0.0047 (10)
P4	0.0184 (3)	0.0224 (3)	0.0199 (3)	−0.0047 (3)	0.0007 (2)	−0.0025 (3)
F4	0.0430 (11)	0.0317 (10)	0.0440 (11)	−0.0200 (9)	−0.0147 (9)	−0.0053 (8)
O4	0.0207 (9)	0.0191 (9)	0.0235 (10)	−0.0036 (7)	0.0035 (7)	−0.0007 (7)
C4	0.0251 (14)	0.0303 (16)	0.0333 (16)	0.0069 (12)	−0.0011 (12)	0.0002 (13)
F5	0.0782 (18)	0.0247 (11)	0.134 (3)	−0.0036 (11)	−0.0712 (19)	−0.0002 (13)
O5	0.0186 (9)	0.0223 (10)	0.0232 (9)	−0.0063 (8)	−0.0033 (7)	−0.0020 (7)
C5	0.0250 (14)	0.0236 (15)	0.054 (2)	−0.0052 (12)	−0.0060 (14)	−0.0057 (14)
O6	0.0338 (11)	0.0214 (10)	0.0461 (13)	0.0031 (9)	−0.0157 (10)	−0.0088 (9)
C6	0.0400 (18)	0.045 (2)	0.0225 (15)	0.0030 (15)	−0.0028 (13)	0.0042 (13)
C7	0.0243 (14)	0.0352 (17)	0.0403 (18)	−0.0036 (13)	−0.0085 (13)	0.0055 (14)
C8	0.0299 (15)	0.0247 (15)	0.0313 (15)	−0.0071 (12)	−0.0065 (12)	0.0019 (12)
C9	0.050 (2)	0.0398 (18)	0.0264 (16)	−0.0216 (16)	−0.0047 (14)	−0.0079 (13)
C10	0.0279 (14)	0.0259 (15)	0.0324 (16)	0.0015 (12)	0.0018 (12)	−0.0050 (12)
C11	0.0315 (15)	0.0391 (18)	0.0253 (15)	−0.0034 (13)	−0.0051 (12)	−0.0055 (13)
C12	0.0236 (14)	0.0383 (17)	0.0307 (16)	−0.0140 (13)	0.0073 (11)	−0.0090 (13)
C13	0.0235 (13)	0.0191 (13)	0.0255 (14)	−0.0069 (11)	−0.0078 (10)	0.0009 (10)
C14	0.0246 (13)	0.0229 (14)	0.0261 (14)	−0.0043 (11)	−0.0096 (11)	−0.0013 (11)
C15	0.054 (2)	0.0300 (17)	0.0333 (17)	−0.0229 (16)	−0.0176 (15)	0.0027 (13)
O7A	0.059 (4)	0.062 (4)	0.029 (3)	−0.007 (3)	−0.003 (2)	−0.032 (2)
O8A	0.030 (2)	0.030 (4)	0.036 (3)	−0.0178 (18)	0.0070 (18)	−0.001 (2)
C16A	0.023 (3)	0.028 (4)	0.015 (3)	−0.0121 (19)	−0.003 (2)	−0.0083 (18)
C17A	0.038 (2)	0.027 (2)	0.0363 (18)	0.0004 (15)	−0.007 (2)	−0.0156 (17)
F6A	0.0312 (16)	0.0262 (15)	0.0452 (17)	0.0042 (12)	−0.0056 (13)	−0.0053 (13)
F7A	0.045 (2)	0.049 (2)	0.0245 (14)	−0.0102 (17)	−0.0080 (12)	−0.0096 (13)
C18A	0.036 (3)	0.0291 (18)	0.050 (2)	−0.0039 (18)	−0.016 (2)	−0.0034 (19)
F8A	0.058 (3)	0.108 (4)	0.0394 (17)	−0.005 (3)	−0.0178 (19)	0.025 (2)
F9A	0.0239 (19)	0.071 (3)	0.055 (2)	−0.023 (2)	−0.0081 (18)	−0.007 (2)
F10A	0.046 (2)	0.0196 (18)	0.151 (5)	0.0063 (18)	−0.048 (3)	−0.025 (2)
O8B	0.092 (9)	0.038 (8)	0.069 (9)	−0.038 (6)	0.058 (7)	−0.016 (6)

### Geometric parameters (Å, °)

**Table:**

Cu1—O1	1.9535 (18)	O6—C13	1.220 (4)
Cu1—O4	1.9582 (18)	C6—H6A	0.9800
Cu1—O3	1.965 (2)	C6—H6B	0.9800
Cu1—O5	1.9863 (19)	C6—H6C	0.9800
Cu1—O2	2.1876 (19)	C7—H7B	0.9800
P1—O1	1.5176 (19)	C7—H7C	0.9800
P1—C1	1.780 (3)	C7—H7A	0.9800
P1—C3	1.786 (3)	C8—H8B	0.9800
P1—C2	1.789 (3)	C8—H8A	0.9800
F1—C14	1.346 (4)	C8—H8C	0.9800
C1—H1B	0.9800	C9—H9B	0.9800
C1—H1C	0.9800	C9—H9C	0.9800
C1—H1A	0.9800	C9—H9A	0.9800
P2—O2	1.498 (2)	C10—H10B	0.9800
P2—C5	1.786 (3)	C10—H10C	0.9800
P2—C6	1.787 (3)	C10—H10A	0.9800
P2—C4	1.792 (3)	C11—H11A	0.9800
F2—C14	1.345 (3)	C11—H11C	0.9800
C2—H2A	0.9800	C11—H11B	0.9800
C2—H2C	0.9800	C12—H12C	0.9800
C2—H2B	0.9800	C12—H12A	0.9800
P3—O3	1.511 (2)	C12—H12B	0.9800
P3—C7	1.779 (3)	C13—C14	1.549 (4)
P3—C8	1.782 (3)	C14—C15	1.516 (4)
P3—C9	1.784 (3)	O7A—C16A	1.222 (8)
F3—C15	1.296 (4)	O8A—C16A	1.213 (9)
C3—H3B	0.9800	C16A—C17A	1.572 (8)
C3—H3C	0.9800	C17A—F6A	1.373 (6)
C3—H3A	0.9800	C17A—F7A	1.378 (6)
P4—O4	1.5116 (19)	C17A—C18A	1.499 (6)
P4—C12	1.782 (3)	C18A—F10A	1.328 (6)
P4—C11	1.783 (3)	C18A—F9A	1.348 (5)
P4—C10	1.787 (3)	C18A—F8A	1.339 (7)
F4—C15	1.334 (4)	O7B—C16B	1.191 (14)
C4—H4C	0.9800	O8B—C16B	1.285 (14)
C4—H4A	0.9800	C16B—C17B	1.569 (8)
C4—H4B	0.9800	C17B—F6B	1.379 (6)
F5—C15	1.337 (5)	C17B—F7B	1.378 (6)
O5—C13	1.265 (3)	C17B—C18B	1.498 (6)
C5—H5A	0.9800	C18B—F10B	1.325 (7)
C5—H5C	0.9800	C18B—F9B	1.349 (5)
C5—H5B	0.9800	C18B—F8B	1.339 (7)
			
O1—Cu1—O4	172.44 (8)	P3—C7—H7A	109.5
O1—Cu1—O3	90.48 (8)	H7B—C7—H7A	109.5
O4—Cu1—O3	88.42 (8)	H7C—C7—H7A	109.5
O1—Cu1—O5	91.12 (8)	P3—C8—H8B	109.5
O4—Cu1—O5	87.71 (8)	P3—C8—H8A	109.5
O3—Cu1—O5	162.51 (8)	H8B—C8—H8A	109.5
O1—Cu1—O2	94.54 (7)	P3—C8—H8C	109.5
O4—Cu1—O2	92.99 (7)	H8B—C8—H8C	109.5
O3—Cu1—O2	102.55 (8)	H8A—C8—H8C	109.5
O5—Cu1—O2	94.69 (8)	P3—C9—H9B	109.5
O1—P1—C1	109.87 (12)	P3—C9—H9C	109.5
O1—P1—C3	113.30 (12)	H9B—C9—H9C	109.5
C1—P1—C3	106.69 (13)	P3—C9—H9A	109.5
O1—P1—C2	112.92 (12)	H9B—C9—H9A	109.5
C1—P1—C2	106.63 (13)	H9C—C9—H9A	109.5
C3—P1—C2	107.03 (14)	P4—C10—H10B	109.5
P1—O1—Cu1	131.81 (11)	P4—C10—H10C	109.5
P1—C1—H1B	109.5	H10B—C10—H10C	109.5
P1—C1—H1C	109.5	P4—C10—H10A	109.5
H1B—C1—H1C	109.5	H10B—C10—H10A	109.5
P1—C1—H1A	109.5	H10C—C10—H10A	109.5
H1B—C1—H1A	109.5	P4—C11—H11A	109.5
H1C—C1—H1A	109.5	P4—C11—H11C	109.5
O2—P2—C5	113.83 (14)	H11A—C11—H11C	109.5
O2—P2—C6	111.92 (15)	P4—C11—H11B	109.5
C5—P2—C6	106.86 (17)	H11A—C11—H11B	109.5
O2—P2—C4	112.71 (13)	H11C—C11—H11B	109.5
C5—P2—C4	105.21 (16)	P4—C12—H12C	109.5
C6—P2—C4	105.69 (16)	P4—C12—H12A	109.5
P2—O2—Cu1	130.30 (11)	H12C—C12—H12A	109.5
P1—C2—H2A	109.5	P4—C12—H12B	109.5
P1—C2—H2C	109.5	H12C—C12—H12B	109.5
H2A—C2—H2C	109.5	H12A—C12—H12B	109.5
P1—C2—H2B	109.5	O6—C13—O5	129.8 (3)
H2A—C2—H2B	109.5	O6—C13—C14	117.6 (2)
H2C—C2—H2B	109.5	O5—C13—C14	112.6 (2)
O3—P3—C7	112.50 (14)	F2—C14—F1	105.9 (3)
O3—P3—C8	114.19 (13)	F2—C14—C15	106.8 (3)
C7—P3—C8	107.45 (15)	F1—C14—C15	107.2 (3)
O3—P3—C9	109.04 (14)	F2—C14—C13	111.1 (2)
C7—P3—C9	108.03 (18)	F1—C14—C13	111.7 (2)
C8—P3—C9	105.23 (16)	C15—C14—C13	113.7 (2)
P3—O3—Cu1	133.98 (12)	F3—C15—F4	109.1 (3)
P1—C3—H3B	109.5	F3—C15—F5	107.2 (3)
P1—C3—H3C	109.5	F4—C15—F5	107.2 (3)
H3B—C3—H3C	109.5	F3—C15—C14	111.1 (3)
P1—C3—H3A	109.5	F4—C15—C14	111.5 (3)
H3B—C3—H3A	109.5	F5—C15—C14	110.6 (3)
H3C—C3—H3A	109.5	O8A—C16A—O7A	131.6 (7)
O4—P4—C12	109.79 (13)	O8A—C16A—C17A	114.0 (6)
O4—P4—C11	110.73 (13)	O7A—C16A—C17A	114.5 (6)
C12—P4—C11	107.01 (16)	F6A—C17A—F7A	104.2 (4)
O4—P4—C10	114.59 (13)	F6A—C17A—C18A	106.1 (4)
C12—P4—C10	107.08 (16)	F7A—C17A—C18A	105.5 (4)
C11—P4—C10	107.30 (16)	F6A—C17A—C16A	114.6 (4)
P4—O4—Cu1	134.85 (12)	F7A—C17A—C16A	111.0 (4)
P2—C4—H4C	109.5	C18A—C17A—C16A	114.4 (4)
P2—C4—H4A	109.5	F10A—C18A—F9A	103.9 (4)
H4C—C4—H4A	109.5	F10A—C18A—F8A	110.5 (5)
P2—C4—H4B	109.5	F9A—C18A—F8A	112.9 (5)
H4C—C4—H4B	109.5	F10A—C18A—C17A	111.3 (5)
H4A—C4—H4B	109.5	F9A—C18A—C17A	111.9 (4)
C13—O5—Cu1	111.38 (18)	F8A—C18A—C17A	106.5 (5)
P2—C5—H5A	109.5	O7B—C16B—O8B	125.4 (8)
P2—C5—H5C	109.5	O7B—C16B—C17B	123.0 (9)
H5A—C5—H5C	109.5	O8B—C16B—C17B	111.4 (9)
P2—C5—H5B	109.5	F6B—C17B—F7B	102.7 (4)
H5A—C5—H5B	109.5	F6B—C17B—C18B	106.1 (5)
H5C—C5—H5B	109.5	F7B—C17B—C18B	105.6 (5)
P2—C6—H6A	109.5	F6B—C17B—C16B	111.5 (5)
P2—C6—H6B	109.5	F7B—C17B—C16B	115.0 (5)
H6A—C6—H6B	109.5	C18B—C17B—C16B	114.9 (4)
P2—C6—H6C	109.5	F10B—C18B—F9B	103.9 (5)
H6A—C6—H6C	109.5	F10B—C18B—F8B	110.0 (5)
H6B—C6—H6C	109.5	F9B—C18B—F8B	112.8 (5)
P3—C7—H7B	109.5	F10B—C18B—C17B	112.6 (5)
P3—C7—H7C	109.5	F9B—C18B—C17B	110.3 (4)
H7B—C7—H7C	109.5	F8B—C18B—C17B	107.3 (5)
			
C1—P1—O1—Cu1	−154.13 (15)	F1—C14—C15—F3	169.7 (3)
C3—P1—O1—Cu1	−34.9 (2)	C13—C14—C15—F3	45.8 (4)
C2—P1—O1—Cu1	86.98 (17)	F2—C14—C15—F4	44.8 (4)
O3—Cu1—O1—P1	166.61 (16)	F1—C14—C15—F4	−68.4 (4)
O5—Cu1—O1—P1	−30.81 (16)	C13—C14—C15—F4	167.7 (3)
O2—Cu1—O1—P1	63.98 (16)	F2—C14—C15—F5	164.0 (3)
C5—P2—O2—Cu1	37.4 (2)	F1—C14—C15—F5	50.8 (3)
C6—P2—O2—Cu1	−83.9 (2)	C13—C14—C15—F5	−73.1 (3)
C4—P2—O2—Cu1	157.08 (16)	O8A—C16A—C17A—F6A	−151.9 (6)
O1—Cu1—O2—P2	−163.26 (16)	O7A—C16A—C17A—F6A	27.5 (8)
O4—Cu1—O2—P2	16.18 (17)	O8A—C16A—C17A—F7A	−34.2 (7)
O3—Cu1—O2—P2	105.23 (16)	O7A—C16A—C17A—F7A	145.2 (7)
O5—Cu1—O2—P2	−71.76 (17)	O8A—C16A—C17A—C18A	85.1 (7)
C7—P3—O3—Cu1	−49.3 (2)	O7A—C16A—C17A—C18A	−95.4 (7)
C8—P3—O3—Cu1	73.5 (2)	F6A—C17A—C18A—F10A	172.6 (4)
C9—P3—O3—Cu1	−169.11 (19)	F7A—C17A—C18A—F10A	62.4 (5)
O1—Cu1—O3—P3	−28.23 (18)	C16A—C17A—C18A—F10A	−60.0 (6)
O4—Cu1—O3—P3	159.25 (18)	F6A—C17A—C18A—F9A	56.9 (6)
O5—Cu1—O3—P3	−123.5 (2)	F7A—C17A—C18A—F9A	−53.3 (6)
O2—Cu1—O3—P3	66.53 (18)	C16A—C17A—C18A—F9A	−175.7 (5)
C12—P4—O4—Cu1	140.10 (18)	F6A—C17A—C18A—F8A	−66.8 (5)
C11—P4—O4—Cu1	−102.0 (2)	F7A—C17A—C18A—F8A	−177.1 (4)
C10—P4—O4—Cu1	19.6 (2)	C16A—C17A—C18A—F8A	60.5 (6)
O3—Cu1—O4—P4	41.48 (18)	O7B—C16B—C17B—F6B	−113.4 (14)
O5—Cu1—O4—P4	−121.46 (18)	O8B—C16B—C17B—F6B	61.0 (11)
O2—Cu1—O4—P4	143.97 (17)	O7B—C16B—C17B—F7B	130.3 (14)
O1—Cu1—O5—C13	−99.79 (18)	O8B—C16B—C17B—F7B	−55.4 (12)
O4—Cu1—O5—C13	72.74 (18)	O7B—C16B—C17B—C18B	7.4 (16)
O3—Cu1—O5—C13	−4.6 (4)	O8B—C16B—C17B—C18B	−178.3 (10)
O2—Cu1—O5—C13	165.56 (18)	F6B—C17B—C18B—F10B	60.9 (6)
Cu1—O5—C13—O6	6.3 (4)	F7B—C17B—C18B—F10B	169.4 (6)
Cu1—O5—C13—C14	−173.07 (17)	C16B—C17B—C18B—F10B	−62.8 (8)
O6—C13—C14—F2	10.4 (4)	F6B—C17B—C18B—F9B	−54.7 (6)
O5—C13—C14—F2	−170.1 (2)	F7B—C17B—C18B—F9B	53.8 (7)
O6—C13—C14—F1	128.4 (3)	C16B—C17B—C18B—F9B	−178.4 (6)
O5—C13—C14—F1	−52.1 (3)	F6B—C17B—C18B—F8B	−177.9 (5)
O6—C13—C14—C15	−110.1 (3)	F7B—C17B—C18B—F8B	−69.4 (6)
O5—C13—C14—C15	69.4 (3)	C16B—C17B—C18B—F8B	58.4 (7)
F2—C14—C15—F3	−77.1 (4)		

### Hydrogen-bond geometry (Å, °)

**Table:**

*D*—H···*A*	*D*—H	H···*A*	*D*···*A*	*D*—H···*A*
C3—H3B···O5	0.98	2.57	3.265 (3)	128
C5—H5A···O4	0.98	2.42	3.204 (4)	136
C10—H10B···O3^i^	0.98	2.58	3.266 (4)	127
C1—H1A···F2^ii^	0.98	2.52	3.392 (3)	149
C1—H1B···O6^iii^	0.98	2.47	3.265 (3)	138
C4—H4A···F4^iv^	0.98	2.51	3.482 (3)	170
C4—H4C···F3^ii^	0.98	2.53	3.478 (5)	163
C9—H9A···F7A	0.98	2.44	3.322 (6)	150
C11—H11B···O8A^v^	0.98	2.42	3.289 (7)	147
C12—H12B···O8A^v^	0.98	2.52	3.388 (8)	147

Symmetry codes:  (i) ; (ii) *x*+1, *y*, *z*; (iii) −*x*, −*y*+2, −*z*; (iv) −*x*, −*y*+1, −*z*; (v) *x*−1, *y*, *z*.
